# The Role of Subjective Task Value in Service-Learning Engagement among Chinese College Students

**DOI:** 10.3389/fpsyg.2016.00954

**Published:** 2016-06-23

**Authors:** Yulan Li, Fangfang Guo, Meilin Yao, Cong Wang, Wenfan Yan

**Affiliations:** ^1^School of Psychology, Beijing Normal UniversityBeijing, China; ^2^Beijing Information Science and Technology UniversityBeijing, China; ^3^Beijing Key Laboratory of Applied Experimental Psychology, School of Psychology, Beijing Normal UniversityBeijing, China; ^4^Department of Educational Studies, Purdue University, West LafayetteIN, USA; ^5^Leadership in Education, College of Education and Human Development, University of Massachusetts, BostonMA, USA

**Keywords:** service-learning, Chinese college student, engagement, subjective task value

## Abstract

Most service-learning studies in higher education focused on its effects on students’ development. The dynamic processes and mechanisms of students’ development during service-learning, however, have not been explored thoroughly. Student engagement in service-learning may affect service-learning outcomes and be affected by subjective task value at the same time. The present study aimed to explore the effect of subjective task value on Chinese college student engagement during service-learning. Fifty-four Chinese college students participated in a 9-weeks service-learning program of interacting with children with special needs. Students’ engagement and subjective task value were assessed via self-report questionnaires and 433 weekly reflective journals. The results indicated that the cognitive, emotional and behavioral engagement of Chinese college students demonstrated different developmental trends during service-learning process. Subjective task value played an essential role in student engagement in service-learning activities. However, the role of subjective task value varied with different stages. Finally, the implications for implementing service-learning in Chinese education were discussed.

A body of research provided evidence supporting the effectiveness of service-learning (Eyler and Giles, 1999; Bringle, 2003; Hart and King, 2007). How students benefit from service-learning and what conditions foster their growth and development, however, have not been explored sufficiently (Dunlap, 1998; Wong, 2008; Jones et al., 2012). Previous studies found that engagement in learning played an important role in improving educational effectiveness (Blumenfeld et al., 2005; Skinner et al., 2008; Fredricks, 2011). Similarly, engagement in service-learning processes also could promote both service quality and students’ outcomes simultaneously (Delve et al., 1990; McGuire and Gamble, 2006; Ngai, 2009). Nevertheless, it is unclear how students engage in service-learning cognitively, emotionally and behaviorally. The mechanism linking service-learning and student engagement still needs examining.

In mainland China, most of the current Chinese students who were born under the one-child policy are treated as ‘a little emperor’ (Cameron et al., 2013; Hvistendahl, 2013). They rarely have opportunities to provide service for others (Han, 2003; Luo et al., 2011). When participating in service-learning, they face many challenges such as a role transition from the service recipient to the service provider and authentic problem solving. According to the expectancy-value theory (Wigfield and Eccles, 2000), how students value the meaningfulness of service-learning may influence their engagement. The present study aimed to explore the dynamics of Chinese college students’ engagement in service-learning and to investigate how subjective task value influences student engagement in service-learning.

## Current Practices and Trends in Service-Learning in Higher Education

Service-learning in higher education, defined as the integration of community service within the context of academic curriculum (Bringle and Hatcher, 1996), is a common practice in the United States as well as many other countries. As demonstrated by an annual survey in America, more than half (55%) of the 1,200 universities surveyed require academic service-learning as part of the core curriculum in at least one major (Campus Compact, 2011). Some meta-analysis studies on the benefits of service-learning participation reported that service-learning may increase academic achievements, promote personal and social development, and improve citizenship awareness (Conway et al., 2009; Celio et al., 2011; Yorio and Ye, 2012). Considering a majority of studies focused on the impacts of service-learning on students’ outcomes, more and more researchers suggested to look beyond simple positive results and shed light on the processes that create such results (Eyler and Giles, 2002; Bringle, 2003; Ngai, 2009). For example, researchers found that if students perceived the learning climate in service-learning as autonomously supportive, interesting and valuable, their engagement and development showed greater improvements over the course of the semester (Levesque-Bristol and Stanek, 2009; Levesque-Bristol et al., 2010). Most of the studies on service-learning engagement, however, may be outcome-based rather than process-based. It is necessary to investigate the dynamic processes and the influencing factors in student engagement during service-learning in order to reveal the mechanism of engagement and to provide suggestions for improving service-learning effectiveness.

In addition, most of the research samples reported in literature about service-learning were selected from schools in the United States. In mainland China, however, service-learning is usually viewed as a form of volunteering and the research in this realm is just in its embryonic stage. Most related literature just focused on introducing how service-learning activities were carried out in foreign countries (Zhao and Zou, 2001; Liu et al., 2005; Pan and Yao, 2011), and there were only a few empirical studies in mainland China. Additionally, as the only child in their families, most Chinese students are looked after carefully in daily life by their parents or grandparents. Meanwhile, influenced by the Chinese traditional culture values, such as the belief that “To be a good student now is to be the top of society in the future”, many Chinese parents regard their children getting high academic grades in school, instead of participating community service, as important and push their children to put their full hearts into academic learning (Luo et al., 2011). In service-learning, most students face the challenge of role transition from the person who receives service to the helper who gives service to disadvantaged people. It is informative to understand the features of service-learning with this kind of role transition.

Reflection is considered to be a key element of service-learning (Eyler and Giles, 2002). In general, personal reflections help students understand the surrounding world and link it to what they learned in the class (Hatcher et al., 2004; Vespia et al., 2012). Reflection has been widely accepted as a method of learning from experience because students describe their thoughts, feelings and behaviors in the process of service-learning in reflective journals. Therefore, reflective journals are usually used as one of the tools for exploring and assessing the characteristics of dynamic process as well as outcomes of service-learning (Gelmon et al., 2001; Hubbs and Brand, 2010). In a related vein, we would apply reflective journals as one of the helpful resources for assessing students’ perceptions about the tasks and the engagement in the service-learning process in this study.

## Student Engagement in Service-Learning

Defined as students’ efforts to devote themselves to purposeful academic activities actively and persistently, student engagement is usually classified into three key components or types: behavioral engagement (BE), emotional engagement (EE), and cognitive engagement (CE) (Fredricks et al., 2004; Appleton et al., 2006). BE refers to the student’s effort, attention, and persistence while initiating and participating in a learning activity. EE refers to the student’s affective reactions in learning activities, including the positive (e.g., interest, happiness) and the negative (e.g., boredom, sadness, and anxiety). Lastly, CE refers to the strategies that students use to control and regulate personal cognition and learning (Fredricks et al., 2004).

Student engagement in learning has captured the interest of researchers from disciplines such as educational psychology, developmental psychology, and other related areas for the past 30 years. These previous studies provided evidence that engagement is one of the key factors in solving student problems such as low achievement, increased levels of boredom and dropout rates (Janosz et al., 2008; Fredricks, 2011; Shernoff, 2013). Engaged students do more than involve themselves in learning activities initiatively and persistently, they also have a high motivation to challenge themselves to succeed. Student engagement is considered to be a useful theoretical model for understanding their learning and development.

Likewise, student engagement is also considered to be a crucial determinant of service learning effectiveness (Delve et al., 1990). Service-learning provides authentic and complex tasks and situations that challenge students’ prejudices, previous experiences and perceptions about the world (Eyler and Giles, 1999; Furco, 2010). Faced with numerous problems and conflicts with which students may be unfamiliar, they have to break out of their comfort zones to involve themselves in service-learning activities (Furco, 2010; Fredricks, 2011), and they may undergo four developmental stages during service-learning process, that is, confusion and hesitancy stage, enlightenment, and enthusiasm stage, fluctuation and adjustment stage, and stabilization and routinization stage (Zeng, unpublished; Guo et al., 2016). Facilitating student engagement in service-learning is an important prerequisite for generating healthy outcomes across multiple realms. Studies found that students’ psychological engagement in service-learning activities predicted their changes in community belonging and social responsibility (McGuire and Gamble, 2006; Ngai, 2009). These studies, however, defined participants’ psychological engagement as perceived importance for both providers and recipients, and failed to distinguish between engagement and subjective task value. In fact, students’ perceived task value may provide an important contribution to their engagement in service-learning. Additionally, most research about the influencing factor of engagement remained at the level of logical explanation with less compelling empirical evidence, let alone in a Chinese context. Therefore, it is necessary to empirically investigate the characteristics of student engagement as well as the influencing factors in service-learning in order to shed light on the mechanism and substantial impacts of engagement.

Previous studies indicated that studying multifaceted and dynamic engagement may help us better understand the complexity of students’ experiences in activities (Blumenfeld et al., 2005; Skinner et al., 2008; Fredricks, 2011), which inspired us to focus on the change in the three types of engagement in service-learning. As stated above, reflective journal is used as a research tool. Students describe their actual thoughts and feelings about the service activity (Hatcher and Bringle, 1997), as well as what they have done in journals, which can correspond to three types of engagement. Therefore, reflection is a valid representation of student engagement changes during the process. In addition, after activities, students may write what they think in the reflective journals without any concerns about pleasing the instructors, because the reflections are irrelevant to their course credit and grade. Therefore, analyzing reflective journals could provide accurate, comprehensive information with high ecological validity for service-learning research.

## The Role of Subjective Task Value in Service-Learning

Research in both in-school and out-of-school settings provided evidence that context matters when assessing engagement. Such context may include teachers, task characteristics and learning environments (Skinner et al., 2008; Fredricks, 2011). According to the expectancy-value theory, one of the key components for understanding student learning is subjective task value (Wigfield and Eccles, 2000). Task value, the belief that a task is worth performing (Wigfield and Eccles, 2000), was demonstrated an indirect effect on academic achievement through engagement or efforts (Greene et al., 2004; Cole et al., 2008). Specifically, if students realize the value of a task for themselves, they will spend much time and make efforts to engage in the task (Bong, 2001; Miller and Brickman, 2004; Liem et al., 2008; Eccles and Wang, 2012). Active participation, engagement, and effort are promoted by tasks that are hands-on, heads-on, project-based, integrated across subjective matter, or in other words, intrinsically motivating, inherently interesting, and fun (Skinner and Pitzer, 2012).

Service-learning is the type of integrative learning that occurs both inside and outside the classroom. During service-learning process, students constantly contemplate the connections between the knowledge learned in the classroom and the knowledge gained during their service experience, thus promoting their personal learning (Eyler and Giles, 2002). When students believe that service tasks can promote their learning, they may engage in service-learning activities. Moreover, authentic service-learning activities may enhance students’ interests and the sense of achievement (Hart and King, 2007; Deeley, 2010) when they receive respect and gratitude from the recipients. These positive feedbacks improve students’ perceptions of the task value and increase their persistence in performing the task (Hart and King, 2007; Johnson, 2009). Therefore, we hypothesized in present study that the task value perception could influence students’ engagement in service activity based on the expectancy-value theory. Unfortunately, there were few studies that explore the relationship between engagement and task value in the context of service-learning.

In conclusion, this study was to explore following three questions:

(1) How do Chinese college students engage in service activities emotionally, cognitively, and behaviorally?
(2) What is the relationship between student’s subjective task value and engagement in service activities?
(3) How does subjective task value influence student engagement during the whole process of service-learning?

## Materials and Methods

### Participants

The participants in this study were 54 sophomores who were majoring in psychology and enrolled in the *Psychology of Learning* course. There were 37 females and 17 males, ranging in age from 18 to 22 years old.

### Instruments

#### Engagement Questionnaire

The questionnaire consisted of three sub-scales, i.e., the BE scale, EE scale, and CE scale. The first two sub-scales were adapted from the engagement scale of Skinner et al. (2008), and the last one was adapted from the Motivated Strategies for Learning Questionnaire (MSLQ; Pintrich et al., 1991).

BE (α = 0.77) was assessed with five items that measured the students’ effort, attention, and persistence in service activities (e.g., “I pay attention while doing the activity”). EE (α = 0.81) was measured with six items that indicated students’ feelings during activities (e.g., “When I’m doing the activity, I feel good”). CE (α = 0.85) was measured with 12 items that involved planning (setting goals), monitoring (of one’s comprehension), and regulating (of one’s learning) (e.g., “When I’m doing the activity, I set goals for myself to direct my activities in each period”).

#### Subjective Task Value Questionnaire

This tool was adapted from the MSLQ developed by Pintrich et al. (1991) and included six items that measured the beliefs of students regarding task value to them (α = 0.87), i.e., their judgments of how interesting, useful, and important the course contents or the specific activities were.

All the items in the questionnaires of engagement and subjective task value were translated into Chinese and adapted to the service-learning context. Participants indicated their level of agreement with each statement using a 7-point Likert scale (1 = “strongly disagree” to 7 = “strongly agree”). Based on the results in a previous service-learning study (Zhang, unpublished), these questionnaires were known to have reasonable consistency reliability and structure validity.

#### Reflective Journal

The reflective journals mainly included open-ended questions like “How do you feel about the activity today? What happened in the activity today? Why? What is your plan for the next time?” Students were asked to complete reflective journal entries after each service activity. The current study would take advantage of reflective journals to investigate the developmental characteristics of task value and engagement throughout the service-learning activities. The development of coding rubrics for task value and engagement were as follows.

##### Task value coding rubric

Based on the previous studies on service-learning (McGuire and Gamble, 2006) and the analysis of students’ journals, the task value was categorized into two types: the value perceived to service providers (TV1) and the value perceived to service recipients (TV2). Following Hubbs and Brand’s (2010) recommendations, the journals were rated in terms of both the quality and the quantity of each task value from 0 (e.g., no description about the task value to himself/herself or to service recipients) to 4 (e.g., described a very high task value to himself/herself or to service recipients several times) (see Appendix A).

##### Engagement coding rubric

Similar to the procedure of task value coding rubric development, a coding rubric for student engagement was established. The rubric included three aspects: emotional (i.e., positive and negative), behavioral and cognitive engagement. Having taken both the quality and quantity of engagement into account, journals were rated, respectively, from 0 (e.g., no description of positive or negative emotion) to 4 (e.g., described strong positive or negative emotions more than two times among the many times mentioned) for EE, from 0 (e.g., not any description on using strategies) to 7 (e.g., described all three strategies used specifically and deeply) for CE, and from 0 (e.g., not any description on observation or interaction) to 6 (e.g., described both aspects specifically and deeply) for BE (see Appendix A).

### Procedure

The course of *Psychology of Learning*, a weekly 2-h lecture throughout the whole semester, was designed to integrate off-campus service-learning. The main task of the 54 students in the service-learning program was “applying learning principles gained in the classroom to serve children with special needs”. The research procedures and self-report survey were approved by the ethical committee at the researchers’ university. College students, children with special needs and their parents were informed that they were free to take part in the study and all of them signed informed consent forms for this study. College students were divided into small groups of 3 or 4 members and they collaborated to serve one child with special needs for approximately 2.5 h per week. Before interacting with the child, college students in each group were asked to design a specific service plan based on the knowledge obtained from the course and the information about the child they were going to serve. At each weekend, the college students interacted with the child to help him or her practice some skills such as how to pass a ball or learn a new word. Students were asked to discuss their experiences and write reflective journal entries immediately after every service activity. The service-learning program lasted 9 weeks. All participants completed self-report questionnaires of engagement and subjective task value after the last day of service activity. However, 1.9–37% of data were missing in several students’ reflective journals during service activities because of some unavoidable cause, such as children or students getting sick or having to participate in another important activity which conflicted with the service-learning. In the end, 54 questionnaires and 433 journal entries were collected.

### Data Analysis

To examine the relationship between subjective task value and student engagement in service activity, the data from the self-report questionnaires and reflective journals were analyzed through the following steps.

First, the data from self-report questionnaires of engagement and subjective task value were analyzed to explore students’ engagement level and its relationship with task value.

Second, to explore the developmental characteristics of task value and engagement in different service-learning stages, the coded scores across weeks were averaged to represent the level of perceived task value and engagement of that stage. The validity and reliability of the task value and engagement rubric were examined before this analysis. Two raters who were blinded to the participants’ information coded 72 journals (randomly selected from 8 participants in 9 time service activities) independently. Each reflective journal of every participant received 5 scores, namely, the value perceived to self (TV1), the value perceived to the service recipients (TV2), EE, CE, and BE. The intra-class correlation coefficients (ICCs) were 0.61, 0.60, 0.70, 0.82, 0.55 for TV1, TV2, EE, CE, and BE throughout the nine time services, respectively, indicating that two raters reached moderate to strong agreement (Landis and Koch, 1977; Garcia-Munitis et al., 2006). Therefore, the rest of the students’ journals were divided into two parts randomly and were rated by the two raters separately. Furthermore, the coding rubrics for task value and engagement were also validated through correlations with corresponding questionnaire measures, respectively. The results showed that TV1 and TV2 were correlated with the task value questionnaire significantly (*r* = 0.31, 0.34, *p*s < 0.05), the EE of the journals was correlated with the EE subscale (*r* = 0.48, *p* < 0.05), as was the CE of the journals and the CE subscale (*r* = 0.27, *p* = 0.075), and the BE of the journals and the BE subscale (*r* = 0.32, *p* < 0.05).

In addition, we inspected the pattern of missing data with an independent *t*-test. By comparing the differences in task value and engagement between participants who had submitted all nine reflective journals and those who failed to submit all journals, it was found that there were no significant differences, respectively, for TV1(*t*s = -1.02∼1.24, *p*s > 0.05), EE (*t*s = -0.33 ∼-1.57, *p*s > 0.05), CE (*t*s = -0.63 ∼ 0.80, *p*s > 0.05), BE (*t*s = -0.02 ∼-1.88, *p*s > 0.05) for seven times, except with TV2 where two differences were found in the 4th and 5th time (*t*s = 3.07, 3.72, respectively, *p*s < 0.05). Because there was no significant difference between the complete and the incomplete dataset, it was reasonable to utilize the data with the complete nine journal entries to serve as a whole (King, 2015).

## Results

### Student Engagement and the Role of Task Value

#### Student Engagement

The results from self-reported survey data of the 54 students indicated that the levels of the three types of engagement were relatively high (*M* > 4). The BE was the highest, followed by EE and CE successively (see **Table 1**).

**Table 1:** Descriptive statistics and correlations for variables from self-reported survey.

Variable	Mean	*SD*	1	2	3	4
(1) Subjective task value	5.85	0.78	1			
(2) Emotional engagement (EE)	5.91	0.64	0.63^∗∗^	1		
(3) Cognitive engagement (CE)	4.89	0.60	0.44^∗∗^	0.61^∗∗^	1	
(4) Behavioral engagement (BE)	6.08	0.56	0.41^∗∗^	0.53^∗∗^	0.26^∗^	1

*^∗^*p* < 0.05, ^∗∗^*p* < 0.01.*

#### The Role of Subjective Task Value in Engagement

As shown in **Table 1**, students’ subjective task value was positively related to CE, BE, and EE. The results of regression analysis indicated that task value significantly predicted engagement (β = 0.54, 0.50, 0.38, *p*s < 0.05 for EE, CE and BE, respectively).

### The Role of Task Value in the Dynamic Process of Engagement

**FIGURE 1:**
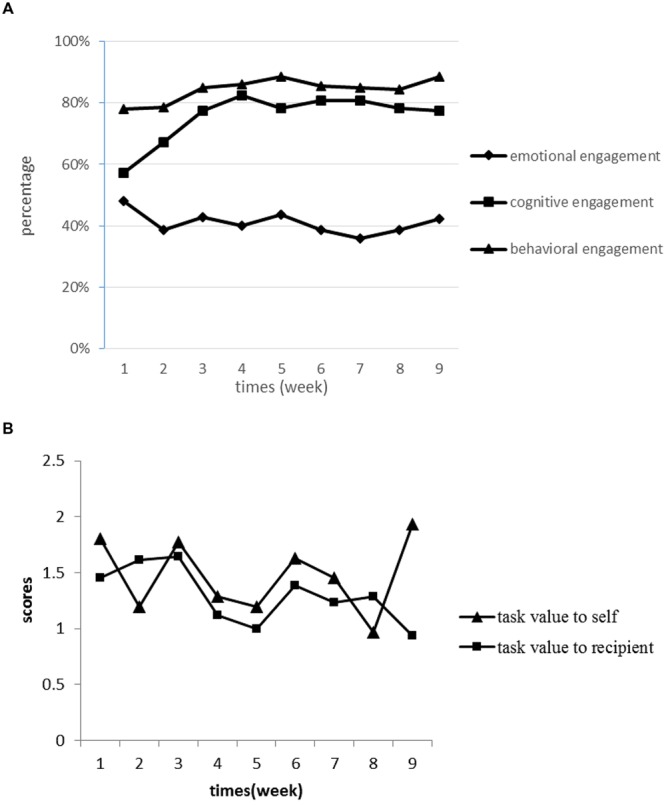
**The Change of Engagement and Subjective Task Value during Service-learning.** Part **(A)** was the changes of three types of engagement. Due to the different scales of emotional engagement (EE), cognitive engagement (CE), and behavioral engagement (BE), the scores were transformed into percentage scores, respectively, for comparison. Part **(B)** was the changes of two types of task value.

To investigate the dynamic characteristics of engagement and the effects of task value, the journals’ data were analyzed. The developmental processes of two types of task values as well as three types of engagement during service-learning were shown in **Figure 1**. It was found that the level of BE was the highest, followed by CE, and EE was the lowest throughout service-learning.

According to our previous studies on service-learning (Zeng, unpublished; Guo et al., 2016), there were four developmental stages of student engagement during nine service-learning sessions: the ‘confusion and hesitancy’ stage (1st time), ‘enlightenment and enthusiasm’ stage (2nd and 3rd time), ‘fluctuation and adjustment’ stage (4th to 7th time), and ‘stabilization and routinization’ stage (8th and 9th time). For the context and implementation of service-learning in the present study was similar to those studies, such stage classification was adopted to describe the development of students’ engagement and the effects of task value.

Based on the above results as well as the qualitative anecdotes from the journals, the developmental characteristics of students’ engagement and the effects of task value were analyzed specifically as follows.

#### The First Stage

As shown in **Figure 1**, students demonstrated a high level of BE and moderate levels of CE and EE. The results also indicated that both types of task value predicted BE significantly, but failed to predict EE and CE significantly (see **Table 2**).

**Table 2:** Summary of regression analysis testing the effect of subjective task value on engagement on four stages.

	1st stage			2nd Stage			3rd stage			4th stage		
	EE	CE	BE	EE	CE	BE	EE	CE	BE	EE	CE	BE
TV1	0.04	0.32	0.44^∗∗^	0.05	0.34^∗^	0.29	0.06	0.44^∗^	0.38^∗^	0.07	0.51^∗∗^	0.25
TV2	0.29	-0.16	0.36^∗^	0.39^∗^	0.32	0.41^∗^	0.31	0.22	0.17	0.07	0.17	0.23
*R*^2^	0.09	0.10	0.40	0.16	0.27	0.31	0.10	0.25	0.18	0.01	0.32	0.13
*F*	1.48	1.60	9.69^∗∗^	2.78+	5.46^∗^	6.46^∗∗^	1.65	4.88^∗^	3.15	0.18	6.78^∗∗^	2.23

*EE, emotional engagement; CE, cognitive engagement; BE, behavioral engagement; TV1, subjective task value to self; TV2, subjective task value to recipient.*

*^∗∗^*p* < 0.01, ^∗^*p* < 0.05, +*p* < 0.10.*

Students perceived high task value to themselves (“*This activity was mutually beneficial rather than providing service only*.”) and engaged behaviorally in this stage. EE was not influenced by task value because students had very high expectations and anxiety during that first experience. They also confronted many situations they had never experienced before when they interacted with the child, which required their extra cognitive regulation [“*At the first time we met, he (the child with special needs) went straight to toy table and ignored our existence, even though we tried hard to communicate with him. We were at our wits’ end. After consulting his parent about the activities he usually enjoys, we have found the good start to interact with him. Gradually, he could say hello and express his positive affection to us. We are so exciting for his progress.*”] (the quotations were taken from the first reflective journal entry of case A22).

#### The Second Stage

As shown in **Figure 1**, students’ EE showed a downward trend from the high starting point in the first stage, while both CE and BE rose steadily. The perceived task value to themselves predicted students’ CE significantly during this stage. The task value perceived to the service recipient affected students’ EE and BE (see **Table 2**).

Having reflected on their gains from service-learning activity [*“It seemed that we accompanied him (the child with special needs). However, he also accompanied us in fact … When interacting with him, I gradually understood that it was important and warm to approve of and encourage a person”*], students’ deep and accurate understanding about the service activity were facilitated (“*The learning goal we set for the child was beyond his ability. He was eager to be encouraged and praised*.”) (excerpted from the second reflective journal entry of case A15). Such statements revealed that perceived task value to themselves could increase cognitive regulation. If students realized the value of the task to the service recipient, they experienced more emotions and were more persistent in service activities. As students indicated in their reflections, their sense of self-efficacy and meaningfulness could be enhanced if their efforts were effective for the recipients [*“I felt a sense of accomplishment and satisfaction when he (the child with special needs) showed his concentration on finding the blocks with same number. Even the slightest progress he made may bring me great pleasure. Though the activity outcome was not as good as what we expected sometimes, I would not give up.*”] (excerpted from the second reflective journal entry of case A25).

#### The Third Stage

All of the three types of engagement demonstrated a trend of fluctuation during this stage (see **Figure 1**). Students’ perceived task value to themselves predicted their CE and BE significantly. The task value to the service recipient did not predict students’ engagement significantly (see **Table 2**). This implied that if students realized the task value for themselves, they would perform more cognitive regulation and be more persistent in service activities.

As students were becoming more and more acquainted with the child, they tried to apply what they learned in class (“*At the beginning, I tried to help the child understand the concept of ‘he’ through establishing S-R association*”), and then constantly monitored and regulated their plan (“*We tried to find out an effective method to teach the child social language all the time…*”) (excerpted from the sixth reflective journal entry of case A19). Unfortunately, realizing the meaning to the service recipient did not lead to high engagement, because students’ feelings fluctuated greatly in this stage [“*Repeating the same training task led Nan (the child with special needs) to some extent of boredom. I had the same feeling as Nan, even though I knew repetition is essential for him to learn a skill. I felt in the dark on how to speed up his learning process. Fortunately, all of us tried our best to adjust the emotions during activity.*”] (excerpted from the forth reflective journal entry of case A7).

#### The Fourth Stage

Students’ EE and BE rose steadily while CE remained stable (see **Figure 1**). Participants in this stage were typically accustomed to the interaction with the children with special needs, and the process became routine for them. Moreover, because the whole service program was drawing to an end, participants tried to make sure that the last services ended with a relaxing atmosphere [“*For the coming activity is the last one, we are going to design an activity for Zhe (the child with special needs) to have fun rather than training his logical thinking as before. At the same time, we should prepare him to accept the fact that we may say good bye to him.*”] (excerpted from the eighth reflective journal of Case A34). Perceived subjective task value to themselves predicted students’ CE only, while the task value to service recipients did not predict students’ engagement significantly (see **Table 2**).

In conclusion, the data from reflective journals provided deep and detailed information about the effects of the subjective task value on students’ engagement in service-learning process. Two types of task value played different roles in different stages. The task value that students perceived for themselves affected engagement throughout the whole service-learning activity, while the task value perceived for the service recipient had greater effects in the early stages than in the later stages.

## Discussion and Implications

While there have been numerous studies related to service-learning in higher education, most of them have focused on the effects of service-learning through one-shot assessments after activities. Consequently, less attention has been paid to the outcomes garnered from the dynamic process of service-learning. As the tasks in service-learning programs are so challenging, student engagement in service-learning is of significant importance. However, there have been few studies that focused on the developmental characteristics of student engagement as well as the influencing factors in the context of service-learning, let alone in the context of mainland China. This study explored the role of subjective task value in engagement of Chinese college students in a service-learning program and identified the different roles of two types of task value in the different stages of service-learning. The mixed methodology of survey and content analysis of reflection was employed. The survey provided a general picture of students’ engagement in service-learning as well as the effects of subjective task value, while the coding data of reflective journals provided specific information about the dynamic characteristics of students’ engagement and the different roles of task value in different service-learning stages. By expanding the task value to egoism and altruistic approaches in the process, this study made a unique contribution to expectancy-value theory. Furthermore, this research deepened the studies on student engagement by investigating how students engage in service-learning, which plays the pioneering role of applying a student engagement paradigm in the classroom to service-learning.

### The Effects of Task Value on Engagement

This study examined the relationship between subjective task value and engagement with the survey data and then illustrated the effects of different kinds of task value on engagement in the process of the service activity based on the data from reflective journals. As Eccles and Wigfield (2002) noted, subjective task value is an important incentive for task engagement. Our findings lend support to the applicability of expectancy-value theory (Jones et al., 2015; Ball et al., 2016) through showing that subjective task value played an important role in engagement in service-learning. Students made contributions to others and constantly thought about the connections between the knowledge learned in class and what they learned as part of their service-learning activities. When the students perceived value from the task, they became fulfilled and competent and were inclined to engage in the task persistently (Johnson, 2009). Even when the learning activities conflicted with what they had learned or with the perceived task value, this kind of cognitive conflict could still promote engagement and cognitive development, according to Piaget’s theory of cognitive development.

Most of the studies on subjective task value mainly emphasize the perceived task value to learners themselves. However, service-learning is a reciprocal activity that benefits both oneself and others. Accordingly, the task value may include two aspects, i.e., the value to oneself and the value to others. The task value perceived to service providers could strongly influence CE and BE, which is consistent with the prior studies (Greene et al., 2004; Jones et al., 2015), while value to service recipients could strongly affect emotional and BE. That is, if students were aware that the task value was more beneficial to them, they would be more persistent, and they would apply more cognitive strategies during the course of the service activity. If students realized the task was beneficial to the service recipient, they would experience many positive emotions such as self-actualization, self-efficacy, and happiness, as well as engagement behaviorally in service activities.

Furthermore, the perceived task value may play different roles in different stages. Specifically, identifying the task value in the second stage appears to be of significant importance. This is because students felt excited the first time they participated in a service-learning activity, and thus, the effect of subjective task value on engagement was limited. Similarly, in the last stage, engagement became habituated and was therefore no longer vulnerable to the influence of task value.

There are some implications for service-learning organizers and practitioners. First, students’ task value should be paid attention to and be increased through well-designed service-learning activities. Appropriate conditions or support should be provided at different stages in order to make good use of different types of task value to increase students’ engagement. Second, both types of task value are meaningful and should be treated with respect. Educators should guide students to think about what service-learning means to themselves (egoism approach) as well as to the service recipients (altruistic approach) and increase students’ efforts, attention, and persistence in service-learning tasks.

### The Features of Different Types of Engagement

Whereas most researchers examined either one type of engagement or combined types of engagement, the present study explored the developmental features of three types of engagement in the context of service-learning in China. The results demonstrated that the participants’ three types of engagement were at a relatively high level even under difficult and challenging circumstances. Specifically, students’ EE fluctuated throughout the service-learning program, while cognitive and BE showed a relatively stable increase. Though students participating in the service activity sometimes felt tired and frustrated, they demonstrated self-control and regulation to meet the demands of the service-learning. Previous studies regarding classroom engagement demonstrated a consistent pattern among three types of engagement (Fredricks, 2011). This study, however, found that the patterns of CE and BE differ from the pattern of EE.

The difference in the findings may be related to the characteristics of service-learning in which students’ positive emotions and negative emotions were often mixed together when they interacted with the children with special needs in an authentic context. Faced with the challenging tasks, students’ emotions were likely to fluctuate. To provide better service to the recipients, students had to continuously adapt their behaviors and adjust the cognitive strategies being implemented. These findings suggest that future research on service-learning should consider different types of engagement, especially EE, and provide effective support for students to devote their knowledge, feelings and behaviors to achieving meaningful and fruitful outcomes.

It is noteworthy that there was an inconsistent result on EE. The data from the survey showed a relatively high EE, while the data from reflective journals were relatively low. The results from the survey revealed the general level of EE and the reflective journals revealed the dynamic changes in EE. It was evident from the quantitative data and the qualitative anecdotes in students’ journals that students experienced mixed emotions such as happiness, helplessness and frustration when they interacted with their service recipients, which led to the fluctuation of their EE. It may reflect the fact that students strove to regulate their cognitive strategies and behavior to better serve their recipients during the process of service-learning, and paid less attention to their feelings. However, students still had a high level of EE when they recalled their service experience and reported on it in the survey. Such results may be explained by the emotional memory theory, which states that emotional information is often remembered and retrieved better than neutral information (Bradley et al., 1992; Dolcos and Cabeza, 2002). That is, students may have positive recollections of service-learning and themselves after experiencing authentic service-learning, which promotes their EE at a higher level.

### The Positive Effect of Service-Learning in Various Contexts

Because there was limited empirical research in a Chinese educational context, the present study tried to explore how Chinese college students engage in service-learning and to investigate the effects of service-learning in different countries. In China, most college students were deeply influenced by the belief that “Only book-learning is exalted” and were brought up being taught to disregard external trivialities and be engrossed in learning. China’s one child policy has exerted more pressure on students to perform well on exams and to succeed in academic learning (Johnson et al., 2007). In this case, Chinese students might put high value on their academic gains and lack consciousness with regard to helping others and providing service persistently. When encountering challenges in service-learning, they were expected to be disengaged during the course of service-learning. In fact, the present study found that their BE increased steadily even under the difficult and challenging circumstances, which was not consistent with the findings in the United States, where participants’ motivation decreased during service-learning in a semester (Darby et al., 2013). The reason for relatively high engagement for Chinese college students may stem from the essential role that subjective task value plays in service-learning. Students may be motivated to participate in service-learning at the beginning by academic goals. When involved in service activities, students tried to build a connecting bridge between classroom learning and their applications in the real world. The improvements in learning outcomes provided positive feedback for students to recognize the task value of their learning. At the same time, students gradually realized that their strengths made a difference and provided value for their service recipients. Both perceived value to themselves and to the recipients helped students construe meaning from their experiences in service-learning. Students considered service tasks as something important and worth pursuing, and then devoted themselves to such an activity. In other words, both charitable actions and academic gains may affect students’ meaning construction about service-learning and broaden their perceived value to not only concern their academic gains but also the welfare of others. This finding in a Chinese context provided additional support for integrating service-learning into school curriculum systems to promote students to develop multiple task values and engage in academic learning and community service. No matter what type of task value there is, it should be realized that subjective task value is one of the essential motivators for engaging in service-learning. The particularly important implication for Chinese education is that any requirements for students participating service activity should take their subjective task value into account.

### Limitations and Future Studies

There are some limitations to the present study. First, we failed to explore other factors that may influence student engagement, such as task difficulty and social support from tutors and peers, and we are going to investigate the effects of task difficulty and tutor feedback on students’ engagement and problem solving in the next study. Second, the coding rubric in this study remains to be verified in subsequent studies. Additionally, different service-learning programs should be designed to gain a better understanding about engagement and task value.

## Author Contributoins

Conception and design of the study: MY, YL, and FG. Acquisition, analysis, and interpretation of data: FG, MY, CW, and WY. Drafting the work and revising it critically for important intellectual content: YL, FG, and MY.

## Conflict of Interest Statement

The authors declare that the research was conducted in the absence of any commercial or financial relationships that could be construed as a potential conflict of interest.
