# Supplementary material for: The Role of Subjective Task Value in Service-Learning Engagement among Chinese College Students
Source: Front Psychol. 2016 Jun 23;7:954. doi: 10.3389/fpsyg.2016.00954 (PMC4917539; doi:10.3389/fpsyg.2016.00954)
Supplement: Supplementary file 1 [file Data_Sheet_1.DOCX]

**Appendix A. The coding rubric for students’ subjective task value and engagement in service-learning activities**

| **Variables** | **Dimensions** | **Content** | **Score** |
| --- | --- | --- | --- |
| Subjective task value | The task value that service provider perceived to himself/herself  (TV1) | The service provider perceived the task is valuable to himself/herself, such as enriching experience, facilitating knowledge understanding and utilization, enhancing self-efficacy, improving critical thinking, or drawing strength from activities, etc. | 0, no any description about the task value to himself/herself;  1, described a little task value to himself/herself once;  2, described a high task value to himself/herself once or a little value more than once;  3, described a very high task value to himself/herself once among the many times mentioned;  4, described a very high task value to himself/herself for several times. |
|  | The task value that service provider perceived to service recipient  (TV2) | The service provider perceived the task is valuable to the service recipient who may learn new skills and habits, make great progresses, enjoy interactive activities with their help. | 0, no any description about the task value to the service recipient;  1, described a little task value to the service recipient once;  2, described a high task value to the service recipient once or a little value more than once;  3, described a very high task value to the service recipient once among the many times mentioned;  4, described a very high task value to the service recipient for several times. |
| Engagement | Positive Emotional Engagement | Student’s positive (e. g., enthusiasm, interest, enjoyment, happiness, warmth, confidence, surprise) emotional involvement during service activities | 0, no any description on positive emotion;  1, described a weak positive emotion only once;  2, described a strong positive emotion once or weak positive emotions more than once;  3, described a strong positive emotion once and weak positive emotions many times;  4, described strong positive emotions more than two times among the many times mentioned. |
|  | Negative Emotional Engagement | Student’s negative (e.g., pressure, anxiety, confusion) emotional involvement during service activities | 0, no any description on negative emotion;  1, described a weak negative emotion only once;  2, described a strong negative emotion once or weak negative emotions more than once;  3, described a strong negative emotion once and weak negative emotions many times;  4, described strong negative emotions more than two times among the many times mentioned. |
|  | Cognitive engagement | The strategies that student used to control and regulate his/her own cognition and learning during service-learning activity; including three aspects: planning, monitoring and regulating | 0, no any description on using strategies;  1, described one of the strategies used , but not specifically or deeply;  2, described one of the strategies used specifically or deeply;  3, described one of the strategies specifically and deeply, or described two of the strategies used with no specification or depth;  4, described two of the strategies used, but one of the two with no specific or deep description;  5, described two of the strategies used specifically and deeply, or described all three strategies used but two of them with no specification or depth ;  6, described all three strategies used and two of them with specification and depth;  7, described all three strategies used specifically and deeply; |
|  | Behavioral Engagement | Students’ effort, attention, and persistence in the service activities,  Including two aspects respectively: observation of and interaction with service recipient | 0, no any description on observation or interaction;  1, described one of the two aspects, but not specifically or deeply;  2, described one of the two aspects specifically or deeply;  3, described one of the two aspects specifically and deeply, or described both aspects with no specification or depth;  4, described both aspects, but one of the two with no specific and deep description;  5, described both aspects, but one of the two with no specific or deep description;  6, described both aspects specifically and deeply. |
